# Highly Transparent and Zirconia-Enhanced Sol-Gel Hybrid Coating on Polycarbonate Substrates for Self-Cleaning Applications

**DOI:** 10.3390/ma16083138

**Published:** 2023-04-16

**Authors:** Bing Zhang, Ruohan Xia, Yao Yan, Jia Liu, Zisheng Guan

**Affiliations:** College of Materials Science and Engineering, Nanjing Tech University, 30 South PuZhu Road, Nanjing 211816, China; 202061203152@njtech.edu.cn (B.Z.); 202061203203@njtech.edu.cn (R.X.); 202061203187@njtech.edu.cn (Y.Y.); 202061203272@njtech.edu.cn (J.L.)

**Keywords:** ZrO_2_-SiO_2_ hybrid coating, hydrophobicity, antireflective, polycarbonate substrates

## Abstract

To improve the efficacy of polymer-based substrate hybrid coatings, it is essential to simultaneously optimize mechanical strength and preserve the optical properties. In this study, a mixture of zirconium oxide (ZrO_2_) sol and methyltriethoxysilane modified silica (SiO_2_) sol-gel was dip-coated onto polycarbonate (PC) substrates to form zirconia-enhanced SiO_2_ hybrid coatings. Additionally, a solution containing 1H, 1H, 2H, and 2H-perfluorooctyl trichlorosilane (PFTS) was employed for surface modification. The results show that the ZrO_2_-SiO_2_ hybrid coating enhanced the mechanical strength and transmittance. The average transmittance of the coated PC reached up to 93.9% (400–800 nm), while the peak transmittance reached up to 95.1% at 700 nm. SEM images and AFM morphologies demonstrate that the ZrO_2_ and SiO_2_ nanoparticles were evenly distributed, and a flat coating was observed on the PC substrate. The PFTS-modified ZrO_2_-SiO_2_ hybrid coating also exhibited good hydrophobicity (WCA, 113°). As an antireflective coating on PC, with self-cleaning capability, the proposed coating has application prospects in optical lenses and automotive windows.

## 1. Introduction

Transparent engineering plastics have gained considerable interest due to their affordability, robust impact resistance, and lightweight nature [1]. The demand for polymer materials to replace traditional oxide glass has risen considerably in a variety of industries, such as transportation and electronics [2,3]. Polycarbonate has emerged as the material of choice for an extensive range of engineering applications, such as photovoltaic devices [4,5], display panels, optical lenses [6,7], optical discs, security windows, and automotive glass [8,9]. Compared with other inorganic transparent materials, such as glass, polycarbonate (PC) offers particular advantages in terms of reduced manufacturing costs and product weight. Despite its high fracture resistance and impact strength, PC has several drawbacks such as low ordinary transmittance (below 90%), poor resistance to UV exposure, and vulnerability to scratching [10,11], all of which contribute to reduced service performance and service life [12,13]. As such, the functionalization of the polymer surface has become a crucial step [14,15]. At present, an effective means of improving the performance and multifunctionality of PC products to meet the increasingly complex environmental requirements is achieved through plating or coating the surface of PC products with organic, inorganic, or hybrid film layers, utilizing various film-forming processes [16]. As energy automobiles and optical devices have developed, there has been increasing demand for PC with self-cleaning and antireflection, which can be realized through different coating technologies. Such methods include several coating methods, namely vapor phase and vacuum deposition of coating materials by physical vapor deposition (PVD) or chemical vapor deposition (CVD) [17]. Sun et al. reported a robust coating with superhydrophobicity that was modified using 1H, 1H, 2H, and 2H-perfluorooctyltrichlorosilane (PFTS) by depositing porous silica nanoparticles on a PC substrate. The coating is superhydrophobic, exhibiting a water contact angle of up to 155°, and possesses certain oleophobic properties [18]. However, the aforementioned process necessitates sophisticated equipment, has a high cost of implementation, and lacks versatility in practical applications. The preparation of organic-inorganic hybrid coatings by means of sol-gel is an alternative method to the vapor-phase or vacuum deposition methods [19,20]. The sol-gel method is simple in operation, has a low reaction temperature with a shorter reaction time [21], and can produce a variety of materials, including powder with strict control of chemical composition, as well as microshaped synthetic materials such as films, particles, tubes, and blocks [22]. At the same time, the introduction of impurities is avoided in the preparation process, thereby ensuring high purity of the synthetic material. Notably, the performance of the final product can be affected by changing the solvent type, pH, reaction temperature, chelator, molar ratio of the reactant, or type of oxide precursor. For example, in the preparation of ZrO_2_-CeO_2_ powder, the difference in pH and chelating agent resulted in the appearance of soft agglomerates and hard agglomerates; the difference in Hf concentration of the precursor plays a decisive role in the grain size and crystallinity of HfO_2_ thin films [23,24,25]. There is a large number of micropores in gel prepared by means of the sol-gel method, and cracks will appear in the drying process. Such issue can be overcome by using dry control chemical additives (DCCA) [26]. Since plastic substrates are heat-sensitive [27], the coating must be cured at a temperature below its glass transition temperature. As is well known, the sol-gel method has been widely used as a simple, low-cost method for preparing coatings [28,29]. Presently, the most extensively researched hybrid coating is the silicon hybrid coating. Due to the abundance of hydroxyl groups, the contact angle between the coating and water typically falls between 30° and 90°, rendering the coating susceptible to staining. Thus, improving the stain resistance of the coating is also a considerable aspect. Malik reported a coating that was applied by means of the sol-gel dip coating method on PC substrates with a nano-hybrid coating based on GPTMS-silica nanoparticles. Subsequently, the hybrid coatings were modified with PFTS. The modified coating exhibited high transparency (transmittance and reflectance spectra, 91% and 4.9% at 550 nm, respectively), high hydrophobicity (CA, 110°), high hardness (3H), and considerably effective cleaning of dust by water droplets [30]. However, the optical transmittance of the coating remained ordinary and exhibited minimal improvement. ZrO_2_ has garnered attention owing to its high refractive index, hardness, high abrasion resistance, and stability, rendering it suitable for use in transparent, optical, and abrasion-resistant coatings. Liping Liang et al. deposited ZrO_2_ film with high optical properties by means of a simple sol-gel method, demonstrating high feasibility [31]. By adding ZrO_2_ to the coating system, the mechanical strength and robustness of the coating can be enhanced [32,33]. Raffaella Suriano et al. reported the application of a hybrid coating comprising silylated fluorinated polymers, silica, and ZrO_2_ mixture, deposited on a PC substrate using the sol-gel spin method [34]. The results showed that the coating had high scratch resistance, outdoor durability, and stability. However, despite the enhancement of the mechanical strength of PC, the coating greatly affected the optical properties. Sara Lopez de Armentia et al. prepared a sol-gel coating with low wettability, high wear resistance, and transparency by coating a silyl solution containing ZrO_2_ nanoparticles on a stainless steel plate using the dip coating method [35]. In this study, a coating of ZrO_2_ sol mixed with MTES-modified SiO_2_ sol was prepared using the sol-gel method. A hybrid coating was then applied to the PC surface by means of the dipping and lifting method, followed by surface modification to achieve a stable hybrid coating that exhibits anti-reflection properties, durability, robust mechanical strength, sturdiness, and self-cleaning capabilities [36,37,38].

## 2. Experimental

### 2.1. Materials

Polycarbonate (PC, 60 mm × 25 mm × 2 mm) was purchased from a local plastic factory and used as the substrate material. (Nanjing, China). Zirconium oxide sol (15 wt%) was purchased from Anhui Xuancheng Jingrui Co. (Xuancheng, China). Tetraethyl Orthosilicate (TEOS, 98%, AR) was purchased from Macklin Co. (Shanghai, China). Acetic acid (CH_3_COOH, 36%, AR) was purchased from Aladdin Co. (Shanghai, China). Anhydrous EtOH was purchased from Shanghai Lingfeng Co. (Shanghai, China). Methyltriethoxysilane (MTES, 98%, AR) was purchased from Aladdin Co. (Shanghai, China). 3-Glycidoxypropyltrimethoxysilane (GPTMS, 98%, AR) was purchased from Aladdin Co. (Shanghai, China). 1H, 1H, 2H, and 2H-perfluorooctyl trichlorosilane (PFTS, 97%) was purchased from Macklin Co. (Shanghai, China). The water was deionized water with a resistance of 18.25 Ω cm^−1^. All the chemical reagents were used directly without further purification.

### 2.2. Treatment of Substrate Materials

The PC was ultrasonic treated in anhydrous EtOH for 30 min. The PC surface was cleaned by washing with purified water to remove surface dust, and the cleaned PC samples were placed on a stand with the uniform front side facing up and dried in an electric blast dryer at 80 °C for 1 h to remove moisture and solvent. The treated PC sheets were wrapped in non-woven fabric.

### 2.3. Synthesis of Mixed Zirconium Oxide and Silica Sols

First, TEOS, EtOH, AcOH, and H_2_O were mixed together in a beaker with stirring at a molar ratio of 1:35:1:4, in which the volume of EtOH was 20 mL. Then the obtained sol-gel was transferred to a closed beaker and stirred in a water bath at 40 °C for 1 h. To modify the sol-gel, MTES was introduced and added gradually to achieve a TEOS/MTES molar ratio of 1, using a graduated dropper. Subsequently, 0.1 mL of GPTMS silane coupling agent was added, and the mixture was stirred vigorously for 2 h. Under the described conditions, six samples were prepared by adding ZrO_2_ sol in the amounts of 0.25 g, 0.50 g, 1.00 g, 1.50 g, 2.00 g, and 2.50 g, before stirring thoroughly for 3 h to obtain stable ZrO_2_ and SiO_2_ mixed sol-gel samples. Finally, the sol-gel samples were aged at room temperature.

### 2.4. Preparation of Mixed Sol-Gel Coating

The coating was made by means of a simple dip-lift method, in which the pre-treated PC was dipped into the mixed sol using a dip-coating machine, dipped in at 80 mm/min, lifted out, air-dried at room temperature, and then placed in a blast drying oven at 90 °C for 2 h.

### 2.5. Surface Modification of the Hybrid Coating

The mixed coating was modified with PFTS solution to enhance the hydrophobic properties. The PFTS solution was prepared as follows: PFTS (3.5 × 10^−3^ mol) was gradually added to EtOH (1.73 mol) with stirring for 20 min at room temperature. The surface of the mixed coating was then immersed in PFTS solution for 10 min and dried at 90 °C for 1 h.

### 2.6. Characterizations

The surface state of the sample was observed by using a scanning electron microscope (SEM, JSM-IT200, Tokyo, Japan). The surface of the sample was coated with a thin layer of gold to improve the conductivity before the test. The chemical composition of the hybrid coating surface was analyzed using X-ray photoelectron spectroscopy (XPS, Thermo Scientific K-Alpha, Waltham, MA, USA). The optical transmittance of the hybrid coating was analyzed using a UV-VIS-NIR spectrophotometer (UV-2600, SGLC, Shanghai, China). The surface roughness of the coatings was analyzed by means of atomic force microscopy (AFM, Bruker, Billerica, MA, USA). Water contact angle measurements of the hybrid coatings were taken using a water contact angle analyzer (JC2000, Shanghai, China) at room temperature. Static water contact angle values were measured three times and averaged. The adhesion between the substrate and the coating was evaluated using an acoustic emission test mode with a WS-2005 coating adhesion automatic scratching instrument (Lanzhou, China). The maximum load was 25 N, with a loading speed of 25 N/min.

## 3. Results and Discussion

### 3.1. Optical Property

**Figure 1:**
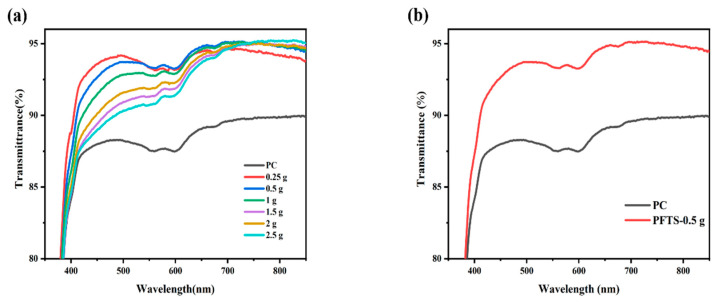
Transmittance spectra of the ZrO_2_-SiO_2_ hybrid coatings (**a**) different contents of ZrO_2_ sol_,_ (**b**) 0.5 g ZrO_2_-SiO_2_ sample modified with 3.5 × 10^−3^ mol PFTS.

Figure 1a shows the transmittance of the coated PC that contained different contents of ZrO_2_. All the coated PC samples exhibited superior transmittance in contrast to the bare PC, and the average transmittance was increased by 3–5%. Such results could likely be attributed to the combined effect of silica with low refractive index and zirconium oxide with high refractive index, which contributes to the refractive index matching between PC and the inorganic particles, thereby minimizing the reflection loss [39,40]. As listed in Table 1, the 0.5 g ZrO_2_-SiO_2_ sample has the best optical property with an average transmittance of 93.9% in the visible wavelength range of 400–800 nm, and a peak transmittance of 95.1%. Comparatively, the peak transmittance of the bare PC is 90.0%. Figure 1b shows the transmittance of the 0.5 g ZrO_2_-SiO_2_ sample after modification with PFTS solution. The results indicate that the transmittance was unchanged and unaffected by the PFTS modification. As such, considering the transmittance and stability of the coating, the PFTS-modified 0.5 g ZrO_2_-SiO_2_ was the optimal sample for the subsequent tests.

### 3.2. Surface Morphology

**Figure 2:**
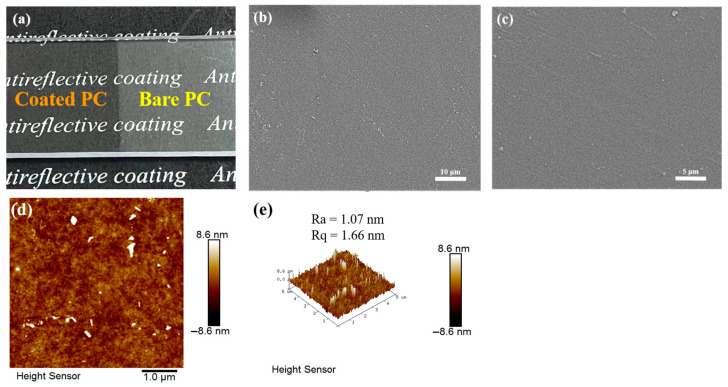
(**a**) Photos of the bare and coated PC, (**b**,**c**) SEM images, and (**d**,**e**) AFM surface topography and 3D images of the PFTS-modified 0.5 g ZrO_2_-SiO_2_ sample.

Figure 2a shows a photograph of the coated PC, evaluated on a macroscopic scale, which demonstrates the antireflective properties of the coated PC. The visual quality of the bare PC is greatly reduced under the effect of light reflection, while the transparency and visual quality of the coated PC are greatly improved, and the text under the PC can be clearly seen through the coated PC. Figure 2b,c shows the SEM images of the PFTS-modified 0.5 g ZrO_2_-SiO_2_ sample. The results indicate that the overall surface of the coating was flat, and there are no obvious abnormal bumps and depressions. Figure 2d,e show the 2D and 3D morphology of the PFTS-modified 0.5 g ZrO_2_-SiO_2_ sample analyzed using AFM. The ZrO_2_ and SiO_2_ nanoparticles were uniformly dispersed throughout most regions of the coating surface, without any instances of agglomeration or phase separation. The number-average roughness (Ra) and mean-square roughness (Rq) of the coating were determined to be 1.07 nm and 1.66 nm, respectively. Such results indicate that the coating surface was substantially flat.

### 3.3. Chemical Compositions

**Figure 3:**
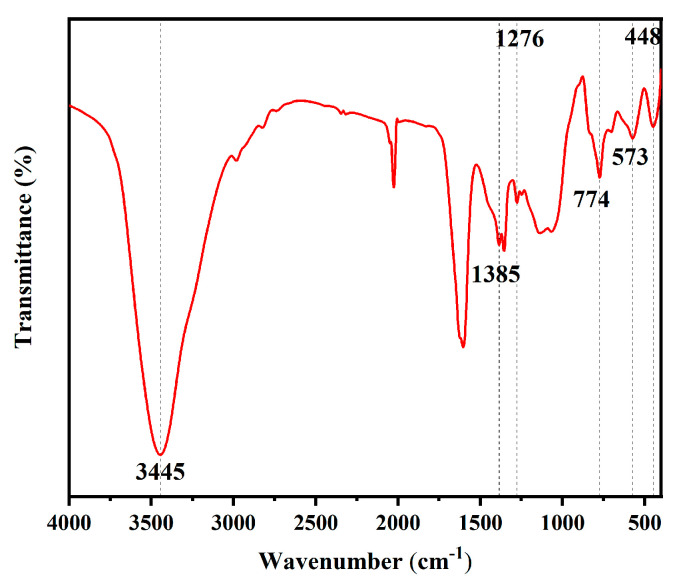
FITR spectra of the PFTS-modified 0.5 g ZrO_2_-SiO_2_ sample.

The FITR spectra analysis of the PFTS-modified 0.5 g ZrO_2_-SiO_2_ sample is shown in Figure 3. The coating showed a characteristic peak at 3445 cm^−1^, which could be ascribed to the stretching vibration of the hydroxyl group on Si-OH, indicating the presence of a large number of hydroxyl groups on the surface; the peak at 1385 cm^−1^ corresponded to the in-plane Si-CH_3_ bending vibration, indicating the incorporation of the MTES. The C-F stretching vibration produced a characteristic peak located at 1276 cm^−1^, which could be attributed to PFTS surface modification. The characteristic peak at 774 cm^−1^ could be ascribed to the Si-O-Si antisymmetric stretching vibration, indicating the hydrolytic condensation during the TEOS as well as the MTES sol-gel process. The peak at 448 cm^−1^ corresponded to the bending vibration of Si-O, while the characteristic peak at 573 cm^−1^ could be ascribed to the vibration of Zr-O, which also indicates the presence of ZrO_2_.

**Figure 4:**
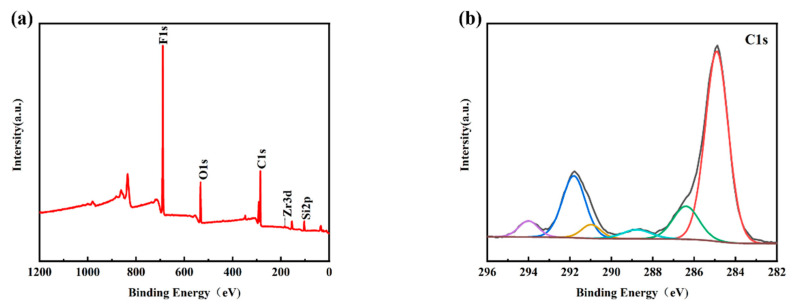

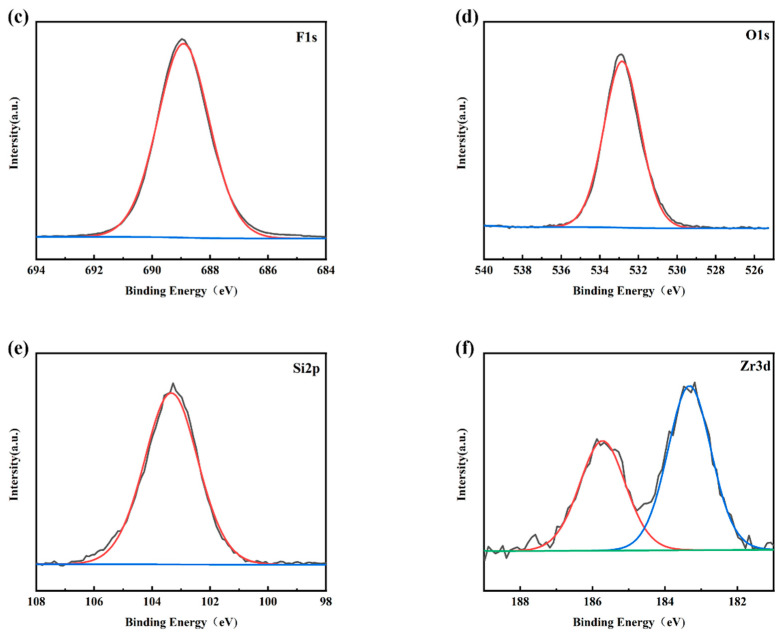
XPS spectra of the PFTS-modified 0.5 g ZrO_2_-SiO_2_ sample. (**a**) XPS survey; (**b**) C1s; (**c**) F1s; (**d**) O1s; (**e**) Si2p; (**f**) Zr3d.

Figure 4a shows the XPS survey spectra of the PFTS-modified 0.5 g ZrO_2_-SiO_2_ sample; the peaks of F, O, C, and Si can be clearly observed. Figure 4b–f shows the high-resolution XPS spectra of C1s, F1s, O1s, Si2p, and Zr3d. For the C1s spectrum, its five peaks are: -C-C (248.80 eV), -C-O (286.29 eV), -C=O (288.65 eV, 290.88 eV), -CF2 (291.80 eV), -CF3 (294.20 eV). As is well known, the presence of silanol (Si-OH) groups leads to a large number of hydroxyl groups (-OH) on the surface of the hybrid coating, and the abundant hydroxyl groups (-OH) also provide conditions for their reaction with PFTS. As shown in the high-resolution spectra presented in Figure 4b,c, the C1s peaks observed at 291.80 eV and 294.20 eV and the F1s peaks observed at 688.2 eV [41] could be attributed to the -CF_2_ and -CF_3_ bonds. Such findings provide clear evidence of the successful modification of PFTS onto the surface of the hybrid coating. The formation of fluorocarbon molecules leads to a low surface area, which corresponds to the low surface energy required to achieve hydrophobicity. Peak 532.74 eV of the O1s spectrum and peak 103.23 eV of the Si2p spectrum correspond to -Si-O. The XPS spectrum of Zr element has two peaks, namely Zr3d5/2 peak at 183.3 eV and Zr3d3/2 peak at 185.7 eV, which correspond to the binding energy of ZrO2 according to the standard spectrum. As shown in Figure 4e,f, the high-resolution spectra of Si2p and Zr3d successfully indicate the hydrolysis of TEOS and the success of the hybrid coating of ZrO_2_.

### 3.4. Wettability, Mechanism of Surface Hydrophobicity, and Self-Cleaning

**Figure 5:**
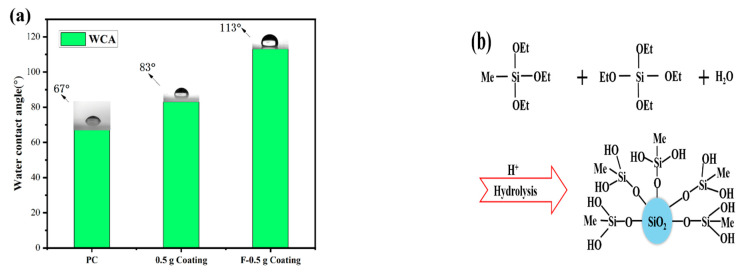

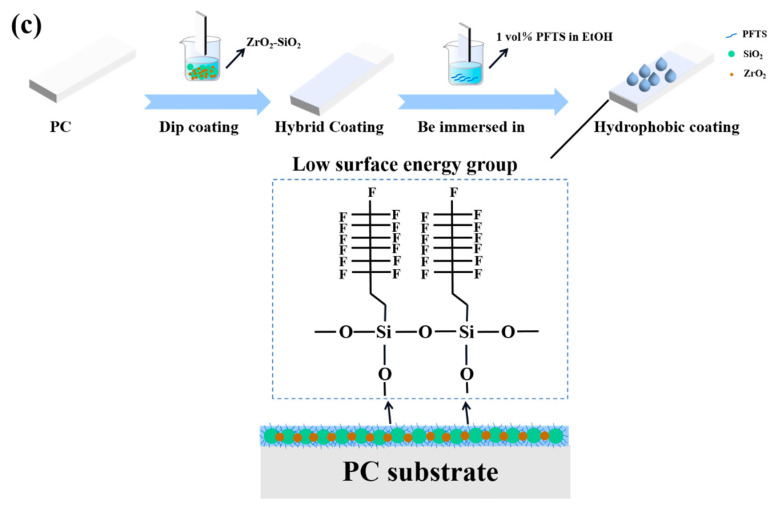
(**a**) WCA of the bare PC, the coating before and after PFTS modification, (**b**) Diagram of MTES/TEOS hydrolysis process, (**c**) Schematic illustration of the mechanism of surface hydrophobicity.

**Figure 6:**
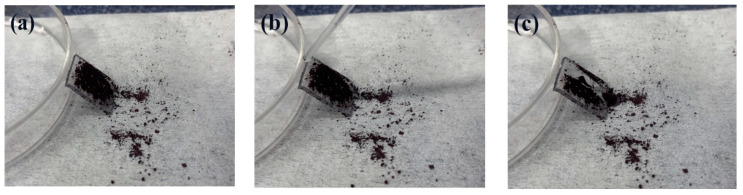
(**a**–**c**) Self-cleaning test for the PFTS-modified sample.

Figure 5a shows the WCA of the coating. Due to the presence of MTES in the coating, several hydrophobic methyl groups will exist on the surface of the coating, as shown in Figure 5b. The WCA of the coating increased from 67° to 83° because of the hydrophobic methyl groups. There were a large number of hydroxyl groups on the surface of the coating, which provided the reaction center for the surface modification. During the modification process of the PFTS solution, the PFTS hydrolyzed and condensed with the hydroxyl group on the surface of the coating to form a low surface energy film, which improves the hydrophobicity (113°) of the coating, as shown in Figure 5c. As shown in Figure 6a–c, the ferric oxide powder was spread on the coating surface. The water droplets rolled on the surface and picked up most of the solid particles to clean the surface, thereby demonstrating that the PFTS-modified coating possesses a certain self-cleaning ability.

### 3.5. Mechanical Stability and Durability

#### 3.5.1. Sandpaper Abrasion Test

**Figure 7:**
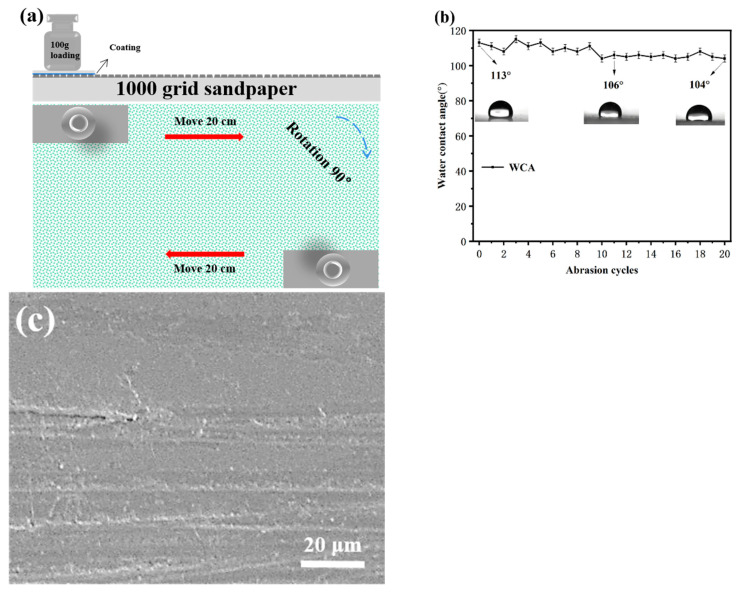
(**a**) Schematic diagram of sandpaper abrasion test, (**b**) variation in WCA with the number of sandpaper abrasion cycles, and (**c**) SEM images of the sample after sandpaper abrasion test.

Figure 7a,b shows the schematic diagram of the sandpaper abrasion test and the variation in WCA on the coated PC after 20 cycles of sandpaper abrasion. As shown in Figure 7b, the coating still had good hydrophobic performance after 20 cycles of sandpaper abrasion, and the WCA was over 100°. Figure 7c shows the SEM image of the coating after 20 cycles of sandpaper abrasion. An observation can be made that the coating suffered some damage to the surface after 20 cycles of abrasion, but there was no large cracking or peeling of the coating. At the same time, the PC surface was protected to a certain extent, which indicates that the coating has good mechanical strength and stability.

#### 3.5.2. Sand Hit Test

**Figure 8:**
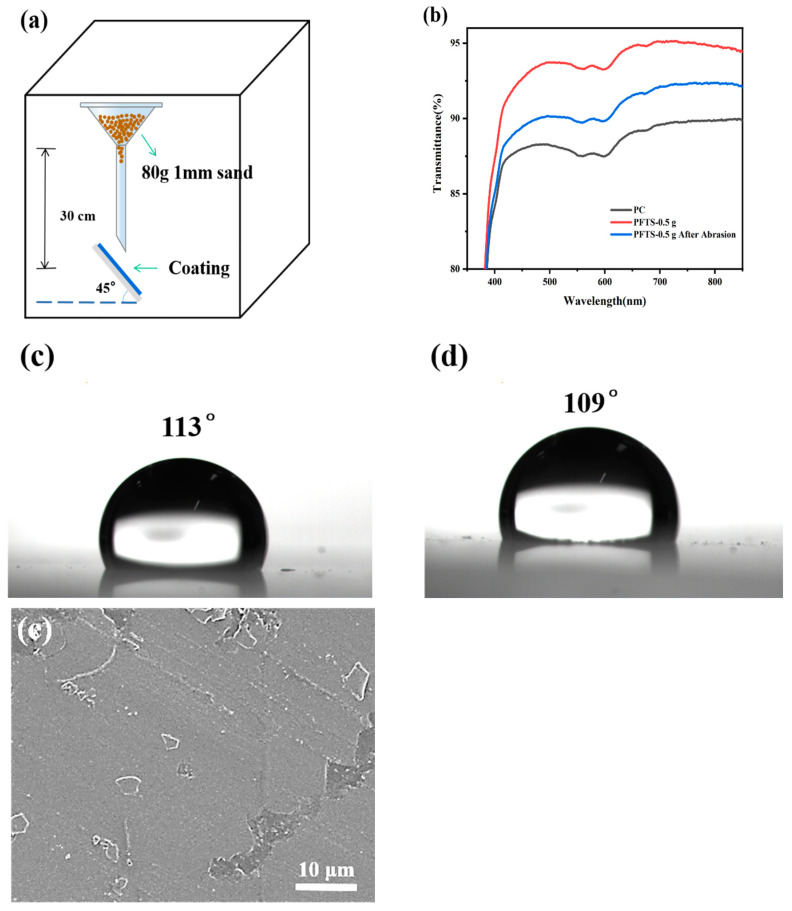
(**a**) Schematic diagram of the sand hit test, (**b**) Transmittance spectra, (**c**,**d**) WCA and (**e**) SEM images of the sample after the sand hit test.

A sand hit test was conducted on the coated PC, and a sand grain with a particle size of about 1 mm was used to hit the coated PC from a height of 30 cm in free fall, as shown in Figure 8a. Figure 8b shows the transmittance of the PFTS-modified 0.5 g ZrO_2_-SiO_2_ sample coated PC after the sand hit test. An observation can be made that although the transmittance was reduced after the sand hit test, the transmittance was still higher than that of the bare PC. Figure 8c,d shows the WCA of the PFTS-modified sample after the sand hit test. An observation can be made that the WCA of the sample did not change greatly, indicating that the coating still had good hydrophobicity. As shown in Figure 8e, a local peeling phenomenon was observed on the coating; however, this did not result in significant cracking or peeling of the coating. Such findings suggest that the coating exhibits a certain degree of stability, robustness, and impact resistance.

#### 3.5.3. Adhesion Scratch Test

**Figure 9:**
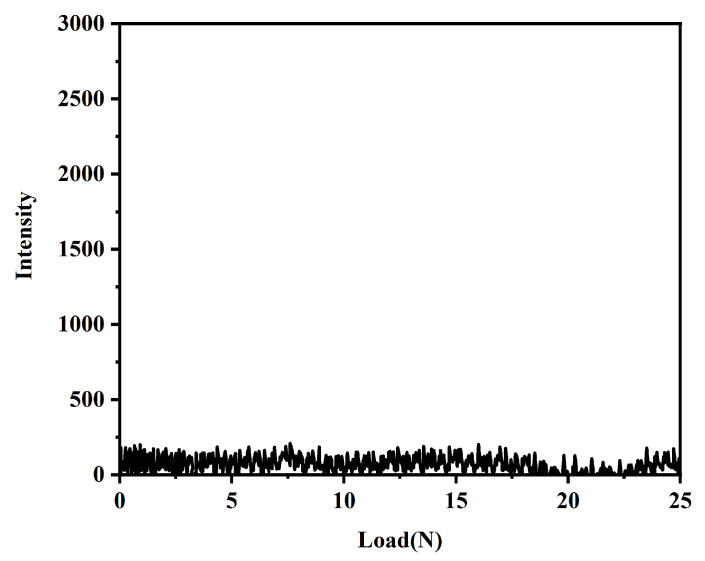
Acoustic emission signals of the PFTS-modified 0.5 g ZrO_2_-SiO_2_ sample (load 25 N, loading time 60 s).

The bonding strength of the coating and the substrate was tested using a coating adhesion auto-scratcher. Figure 9 shows the coating substrate bonding strength of the coating with the acoustic emission signal. As shown in Figure 9, the acoustic emission signal did not change significantly throughout the experiment, indicating that the coating has sufficient bonding strength with the substrate.

#### 3.5.4. Water-Drop Impact Test and Stability Test

**Figure 10:**
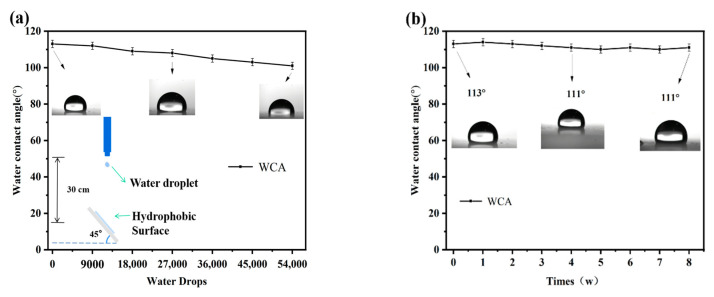
Changes in WCA (**a**) the number of water drops in the free-fall water drop test, (**b**) exposure time to air.

To further investigate the mechanical stability of the hydrophobic coating surface, water-drop impact simulation experiments were conducted on the PFTS-modified 0.5 g ZrO_2_-SiO_2_ sample. The schematic diagram of the water-drop impact test is shown in Figure 10a. The coating maintained hydrophobicity after 54,000 water droplet impacts, indicating that the coating could withstand the test and has good resistance to water droplet impacts. Figure 10b shows the effect of exposure time on the WCA of the surface. The PFTS-modified 0.5 g ZrO_2_-SiO_2_ sample coated PC was exposed to air to check its stability under natural aging conditions. After 8 weeks of exposure to air, the WCA of the coating did not change significantly, indicating that the hydrophobic coating has good durability and stability.

## 4. Conclusions

In conclusion, a mixture of zirconium dioxide(ZrO_2_) sol and methyltriethoxysilane-modified silica(SiO_2_) sol-gel was successfully dip-coated on PC substrates to form ZrO_2_-enhanced SiO_2_ hybrid coatings. Additionally, a solution containing PFTS was employed for surface modification as demonstrated by both FTIR, where characteristic peaks corresponding to SiO_2_ and ZrO_2_ could be observed, and XPS, which indicated the presence and correct valence states of the elements. The PFTS-modified ZrO_2_-SiO_2_ hybrid coatings show high transparency (average transmittance up to 93.9% and peak transmittance up to 95.1% at 700 nm, respectively), hydrophobicity (WCA, 113°), and good mechanical strength. After a series of tests, the coating could still maintain its properties. Therefore, the belief of the present authors is that the PFTS-modified ZrO_2_-SiO_2_ hybrid coatings can contribute to the surface functionalization of other polymer substrates and have promising applications in optical lenses, automotive windows, and self-cleaning antifouling.

## Figures and Tables

**Table 1:** Average and peak (400–800 nm) transmittance of bare and coated PC substrate.

ZrO_2_ Sol (g)	Average Transmittance [%]	Peak Transmittance [%]
Bare PC	88.6	90.0
0.25	93.8	94.7
0.50	93.9	95.1
1.00	93.5	95.1
1.50	92.5	95.0
2.00	92.9	95.0
2.50	92.3	95.0

## Data Availability

Not applicable.
